# Real-Time Object Tracking via Adaptive Correlation Filters

**DOI:** 10.3390/s20154124

**Published:** 2020-07-24

**Authors:** Chenjie Du, Mengyang Lan, Mingyu Gao, Zhekang Dong, Haibin Yu, Zhiwei He

**Affiliations:** 1 School of Electronic Information, Hangzhou Dianzi University, Hangzhou 310018, China; ducj@hdu.edu.cn (C.D.); 182040114@hdu.edu.cn (M.L.); mackgao@hdu.edu.cn (M.G.); englishp@hdu.edu.cn (Z.D.); shoreyhb@hdu.edu.cn (H.Y.); 2 Zhejiang Provincial Key Lab of Equipment Electronics, Hangzhou 310018, China

**Keywords:** correlation filter, histogram of oriented gradient, color naming, dual-template, target re-detection

## Abstract

Although correlation filter-based trackers (CFTs) have made great achievements on both robustness and accuracy, the performance of trackers can still be improved, because most of the existing trackers use either a sole filter template or fixed features fusion weight to represent a target. Herein, a real-time dual-template CFT for various challenge scenarios is proposed in this work. First, the color histograms, histogram of oriented gradient (HOG), and color naming (CN) features are extracted from the target image patch. Then, the dual-template is utilized based on the target response confidence. Meanwhile, in order to solve the various appearance variations in complicated challenge scenarios, the schemes of discriminative appearance model, multi-peaks target re-detection, and scale adaptive are integrated into the proposed tracker. Furthermore, the problem that the filter model may drift or even corrupt is solved by using high confidence template updating technique. In the experiment, 27 existing competitors, including 16 handcrafted features-based trackers (HFTs) and 11 deep features-based trackers (DFTs), are introduced for the comprehensive contrastive analysis on four benchmark databases. The experimental results demonstrate that the proposed tracker performs favorably against state-of-the-art HFTs and is comparable with the DFTs.

## 1. Introduction

Visual object tracking is a challenging job and a hot research topic of the computer vision community. At present, with the technical improvement of hardware facilities and artificial intelligence, visual object tracking plays a critical role in innumerable applications (i.e., human-computer interaction, robot navigation, surveillance systems, handwritten recognition) [1]. Numerous object tracking works [2,3,4,5,6,7] have been widely researched to overcome the difficulty of tracking failures. Generally, object trackers can be divided into either generative trackers [8,9] or discriminant trackers [10,11], depending on whether a model utilizing background information is used to predict the target location. The results for the object tracking benchmark (OTB) [12,13] and visual object tracking (VOT) [14,15] show that discriminant tracking algorithms are superior to generative tracking algorithms in terms of tracking overall performance.

**Figure 1:**
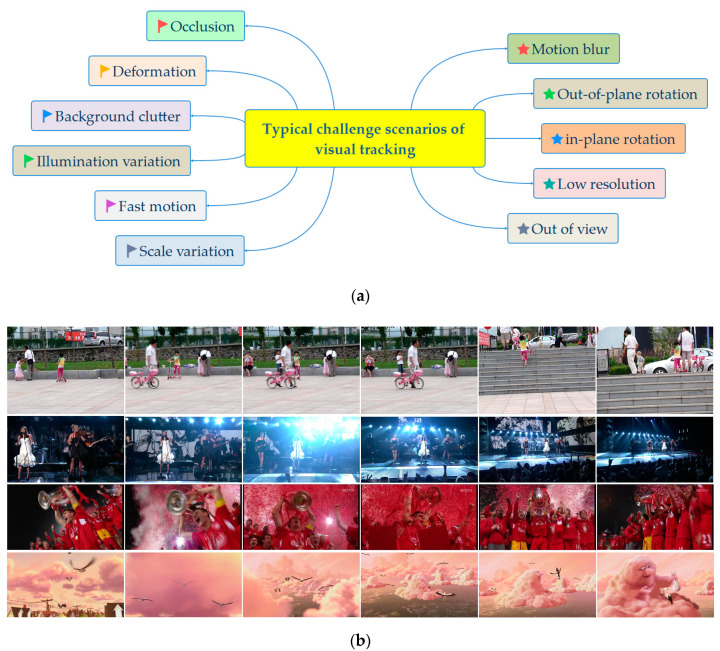
A variety of complicated challenge scenarios of visual tracking: (**a**) the 11 common video challenge scenarios of visual tracking; (**b**) the target objects are in different pose and appearance variations.

Recently, owing to the advantages of simplicity, robustness, and high accuracy, correlation filter-based trackers (CFTs) have received ample attention in visual object tracking [16,17]. Although the CFTs have made great achievements, tracking drifting or even loss may occur due to partial or complete occlusion, motion blur, deformation, illumination variation, and background clutter, etc. It is difficult to improve the performance of the tracking algorithm in different challenge scenarios. A variety of complicated challenge scenarios are shown in Figure 1.

It is challenging to design a robust tracker. The CFT is a typical discriminant tracking algorithm. It evolved from the earliest minimum output sum of squared error (MOSSE) [18] to the circulant structure of tracking-by-detection with kernels (CSK) [19] and the kernelized correlation filter (KCF) [20]. KCF improves performance while ensuring a higher running speed. Learning spatially regularized correlation filters for visual tracking (SRDCF) [21] employ a large detection area and add weight constraints to filter coefficients to effectively alleviate the boundary effect. Generally, in complicated challenge scenarios, the above-mentioned CFTs still experience serious performance decline in the discrimination ability. In [22], multiple features were combined to improve the tracking performance and seek the optimal feature combination. In [23], Alexandros Makris et al. proposed a particle filter based tracking algorithm and fused the features of the salient points and color histogram. This tracker tackled the multimodal distributions emerging from cluttered scenes. Furthermore, most existing trackers suffer from many limitations: (1) the sole filter template is used; (2) the search range of the object is fixed; (3) the fusion weight coefficient is invariable during the updating process.

Additionally, owing to the strong representation ability of deep features, the correlation filter with depth features can achieve great success in visual object tracking, while the heavy computation complexity inevitably results in low tracking speed and limited application in practical systems. Hence, a real-time dual-template CFT for various typical challenge scenarios is proposed in this work. In contrast to existing object tracking algorithms, the main contributions of this paper are as follows:

(1) To solve the limitation of the sole filter template, a dual-template method is proposed to improve the robustness of the tracker;
(2) In order to solve the various appearance variations in complicated challenge scenarios, the schemes of discriminative appearance model, multi-peaks target re-detection, and scale adaptive are integrated into the tracker;
(3) A high-confidence template updating technique is utilized to solve the problem that the filter model may be drift or even corruption.

The rest of this paper is organized as follows. Related work is discussed in Section 2. Then, the classical kernel correlation filter is presented in Section 3. The details of the proposed tracking method are illustrated in Section 4. Section 5 evaluates the performance of the proposed tracking method through experimental simulations. Finally, Section 6 concludes this work.

## 2. Related Work

In this section, several related methods are briefly described, including the early object tracking algorithms, the convolutional neural network (CNN)-based object tracking algorithms, and the correlation filter-based object tracking algorithms.

### 2.1. The Early Object Tracking Algorithms

In [8], texture features were added to the mean shift algorithm for tracking framework, because texture features can better describe the apparent features of the object and overcome the interference of background color similarity. In [24], a heuristic local anomaly factor was proposed to improve the tracking accuracy by using global matching and local tracking methods. Li et al. [25] proposed a patched based tracker by using reliable patches for object tracking. In [26], the scheme of mixed-integer programming was utilized to track a ball in team sports. The above object tracking algorithms are processed in the time domain. In the tracking process, it involves a complex matrix inversion calculation, which has a large amount of calculation and poor real-time performance.

### 2.2. The CNN-Based Object Tracking Algorithms

Hierarchical convolutional features (HCF) [27] reconciled the shallow and deep features of the visual geometry group (VGG) network and integrated them into a correlation filter, which obtained good tracking performance. However, HCF does not consider the scale variation and assumes that the target scale is constant in the whole tracking sequence. The multi-domain network (MDNET) [28] tracking algorithm exploits a small network to learn the convolution features and uses a softmax strategy to classify the samples. The performance of MDNET is excellent, yet the speed is only 1 frame per second (FPS). Adversarial deep tracking (ADT) [29] employs the deep convolutional generative adversarial networks, which are composed of the fully convolutional siamese network (SiamFC) [30] and the classification network. ADT can be trained and optimized end-to-end through adversarial learning. Wang et al. [31] integrated CNN features to the correlation filter framework. The peak-to-sidelobe ratio (PSR) is utilized to measure the differences between image patches. In [32], a sample-level generative adversarial network was used to expand the training samples, and a label smoothing loss regularization can obtain filter model regularization and reduce overfitting, the speed of the tracker is only 1.02 FPS on OTB-2013 dataset. Huang et al. proposed GlobalTrack, which is developed based on two-stage target detectors [33]. The GlobalTrack method can run without cumulative errors and obtain the best performance on four large-scale tracking datasets. In [34], a robust long-term tracking based on skimming and perusal modules (SPLT) was proposed. This robust algorithm ranked first on the VOT2018 long-term dataset. A novel long-term tracking framework based on deep regression and verification networks was proposed by Zhang et al. [35]. The Distractor-aware Siamese Networks [36] exploited an effective sampling strategy to control the distribution of training samples and make the model focus on the semantic distractors. Li et al. proposed the SiamRPN++ approach [37] to take advantage of features from deep networks and improve the accuracy of the tracker. GlobalTrack, SiamRPN++, and DaSiam_LT rank first, third, and fourth in all the long-term tracking approaches at home and abroad, respectively. Generally, as the network layers number of DFTs increases, the computational complexity and the storage space of parameters are increased exponentially.

### 2.3. The Correlation Filter-Based Object Tracking Algorithms

The earliest correlation filter tracker was the MOSSE filter, which was derived from signal correlation and simplified using Fourier correlation properties. Henriques proposed the CSK by introducing dense sampling and exploiting the Fourier diagonalization property of circulant matrices to accelerate the training and detection process. The CSK can run at an average speed of up to 362 FPS on the OTB-2015 dataset. The KCF was proposed based on the CSK by employing the kernel function and extending the multichannel histogram of oriented gradient (HOG). The surface texture feature and contour shape of the object can be well described by the HOG. Danelljan proposed a filter extended with color naming (CN) [38], which can effectively use the color information of the target. To improve the tracking speed, principal component analysis (PCA) is also used to reduce the 11-dimensional features to two-dimensional features. In [39], Zhu et al. presented a tracker that is not limited to a local search window and can efficiently probe the entire frame. In [40], fast discriminative scale space tracking (fDSST) employed a novel scale adaptive tracking method by learning separate discriminative correlation filters, which can improve the robustness and speed of tracker. Gao et al. proposed a maximum margin object tracking with weighted circulant feature maps (MMWCF) [41] to reduce the influence of inaccurate samples. An anti-occlusion correlation filter-based tracking method (AO-CF) [42] proposed an occlusion criterion and a new detection condition for detecting proposals. Long-term correlation tracking (LCT) [43] consists of translation and scale estimation and trains an online random fern classifier to re-detect objects in case of tracking failure. The CFTs possess the following superiorities: (1) CFTs skillfully use fast Fourier transform (FFT) to calculate the correlation characteristics between samples and filter template and find the target area with the greatest output response, making the trained filter model more reliable and accurate; (2) CFTs can transform the multiplication operation between matrices into element point multiplication, which can substantially improve the computation speed and achieve real-time tracking; (3) the tracking accuracy of correlation filter-based trackers can meet the needs of many applications and can be realized on embedded devices.

## 3. Kernel Correlation Filter Tracker

In this section, the principle of the kernel correlation filter tracker is explained in detail. It is noted that the introduction of the kernel correlation filter makes the tracker more robust and can deal with nonlinear classification problems.

### 3.1. Ridge Regression and Utilization of Circulant Matrix

In the kernel correlation filter tracker, Henriques et al. employed the form of ridge regression to solve filter coefficients. The ridge regression is a regularized least square method. Compared with traditional trackers [24,25,26], the ridge regression is more efficient and can obtain a simple closed-form optimal solution. The algorithm aims to learn the filter model from the training image patch *x* with the size of *M* × *N*. At each location (*m*, *n*){0, 1, …, *M* − 1} × {0, 1, …, *N* − 1}, the training samples are generated by the circular shifts of image patch *x_m,n_*. The Gaussian function label *y* = exp{−((*m* − *M*/2)^2^ + (*n* − *N*/2)^2^/2*σ*^2^)}. The objective function is defined as:

(1) $$\min\left(\sum_{i}{\left(f\left(x_{i}\right)-y_{i}\right)}^{2}+\lambda {\left\|\omega \right\|}^{2}\right),$$

where *f*(*x*) = *ω*^T^*x* and *λ* > 0 is a regularization parameter to control overfitting. Then, according to the computing method in [44], *ω* can be calculated directly as:

(2) $$\omega ={\left(X^{T}X+\lambda I\right)}^{-1}X^{T}y,$$

where each element *x_i_* of the matrix *X* represents a sample, each element *y_i_* of *y* represents the regression target value, and *I* is the unit matrix. In the frequency domain, Equation (2) is replaced by the complex form, which is now rewritten as:

(3) $$\omega ={\left(X^{H}X+\lambda I\right)}^{-1}X^{H}y,$$

where *X*^H^ is the Hermitian transpose of *X*.

Through Equation (3), it can be found that the closed-form solution of the model parameter *ω* has the process of matrix inversion. The matrix inversion process will significantly reduce the tracker’s speed, and become an obstacle factor in the realization of real-time target tracking. To solve this problem, the circulant matrix is introduced into the kernel correlation filter tracker. The circular shifts of image patches are used to generate the training samples. To simplify symbols, we assume that the positive sample data are *x* = [*x*_1_, *x*_2_, *x*_3_, …, *x_n_*]^T^. Matrix *P* is called the cyclic translation operator, which can be expressed as:

(4) $$P=\left[\begin{array}{ccccc} 0 & 0 & 0 & \cdots & 1\\ 1 & 0 & 0 & \cdots & 0\\ 0 & 1 & 0 & \cdots & 0\\ \vdots & \vdots & \ddots & \ddots & \vdots \\ 0 & 0 & \cdots & 1 & 0 \end{array}\right].$$

From Equation (4), a cyclic shift of the data can be represented as *Px* = [*x*_n_, *x*_1_, *x*_2_, …, *x*_n-1_]^T^. All the cyclic sample data can be obtained as:

(5) $$\left\{P^{v}x|v=0,1,\ldots ,n-1\right\},$$

where *v* is the number of cyclic shifts. All cyclic sample data can be concatenated as the circulant matrix *X* = *C*(*x*), which is defined as:

(6) $$X=C\left(x\right)=\left[\begin{array}{ccccc} x_{1} & x_{2} & x_{3} & \cdots & x_{n}\\ x_{n} & x_{1} & x_{2} & \cdots & x_{n-1}\\ x_{n-1} & x_{n} & x_{1} & \cdots & x_{n-2}\\ \vdots & \vdots & \ddots & \ddots & \vdots \\ x_{2} & x_{3} & x_{4} & \cdots & x_{1} \end{array}\right].$$

The circulant matrix *X* has many special properties, which can be diagonalized by the discrete Fourier transform (DFT).

(7) $$X=Fdiag\left(\hat{x}\right)F^{H},$$

where *F* means the DFT matrix. It is employed for transforming the sample data to the Fourier domain. *F*^H^ is the Hermitian transpose of *F*, and *diag* means the diagonal matrix. Equation (7) can be regarded as the characteristic decomposition of the cyclic matrix *X*. By employing the property of *X* in Equation (7), the covariance matrix *X*^H^*X* can be computed by:

(8) $$X^{H}X=Fdiag\left({\hat{x}}^{*}⊙\hat{x}\right)F^{H}.$$

Then utilizing the properties of DFT and substituting Equation (8) into Equation (3), we can obtain:

(9) $$\hat{\omega }=\frac{{\hat{x}}^{*}⊙\hat{y}}{{\hat{x}}^{*}⊙\hat{x}+\lambda },$$

where $\hat{\omega }$, $\hat{x}$, and $\hat{y}$ are the DFT of *ω*, *x*, and *y*, respectively. ${\hat{x}}^{*}$ is the complex conjugation of $\hat{x}$, the operator ⊙ denotes the Hadamard product, and *λ* is a regularization parameter for ridge regression.

### 3.2. Kernel Trick

The kernel trick is used to map the linear input to the nonlinear eigenspace and can improve the performance of the tracker. The classifier *f*(*x*) is further transformed into:

(10) $$f\left(x\right)=\omega^{T}\varphi \left(x\right)=\sum_{i=1}^{n}\alpha_{i}\kappa \left(x_{i},x_{i}^{\prime }\right).$$

Then, for most of the kernel functions, the kernel form of the ridge regression is solved as:

(11) $$\hat{\alpha }=\frac{\hat{y}}{{\hat{k}}^{xx^{\prime }}+\lambda },$$

where *k^xx^’^^* represents the first-row element of the kernel matrix *K* = *C*(*k^xx^’^^*), and *k^xx^’^^* is computed by:

(12) $$k^{xx^{\prime }}=\exp\left\{-\frac{1}{\sigma^{2}}\left({\left\|x\right\|}^{2}+{\left\|x^{\prime }\right\|}^{2}-2F^{-1}\left(\sum_{d}{\hat{x}}_{d}^{*}⊙{\hat{x}}_{d}^{\prime }\right)\right)\right\},$$

where *d* denotes the number of channels of the feature layer, *σ* is the parameter of the kernel function, and *F*^−1^ is inverse discrete Fourier transform (IDFT).

### 3.3. Fast Target Detection and Model Update

In the tracking process, the kernel correlation filter tracker extracts the features *x* from the image patch centered on the object location. When the next frame comes, the features *z* are extracted from the image patch in the same way as the previous frame. The tracker calculates the output response of the current frame in the Fourier domain as:

(13) $$f\left(z\right)=F^{-1}\left({\hat{k}}^{xz}⊙\hat{\alpha }\right),$$

where *F*^−1^ is the IDFT, ⊙ is the dot product, and *k^xz^* represents the kernel correlation of training sample *x* and testing sample *z* (i.e., features). The calculated maximum value of *f*(*z*) demonstrates the object position of the current frame. To adapt to the changes of the object appearance model, the filter is updated by utilizing Equation (11) to train the *α*_new_. Then, the updating process of the new filter can be linearly expressed as:

(14) $${\hat{\alpha }}^{t}=\left(1-\gamma \right){\hat{\alpha }}^{t-1}+\gamma {\hat{\alpha }}_{new},$$

where *γ* is the learning rate parameter.

## 4. The Proposed Tracker

In this section, based on the kernel correlation filter tracker, we integrate the dual-template, scale estimation, and adaptive template updating strategy components, and a detailed description of our proposed algorithm is provided.

### 4.1. The Framework of the Proposed Approach

**Figure 2:**
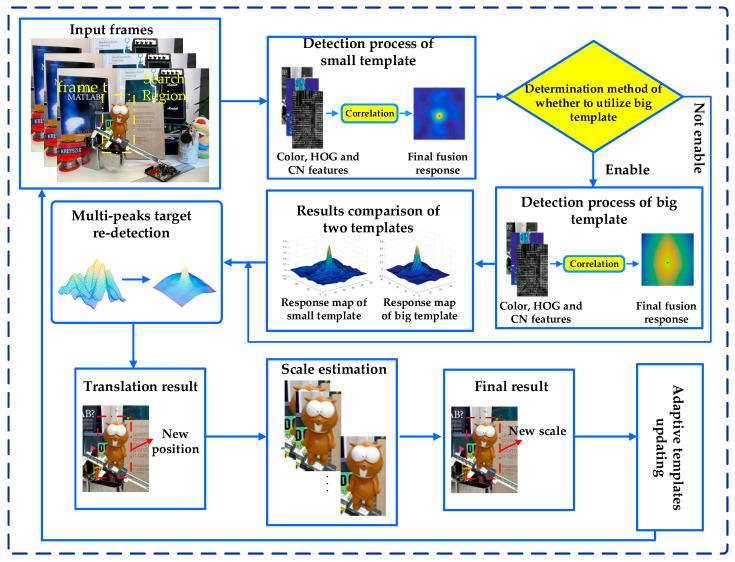
The framework of the proposed algorithm illustrated by the lemming sequence in frame *t*.

In this paper, we propose a novel dual-template CFT. The main difference to the existing tracking algorithms is that our tracker utilizes the strategies of dual-template, discriminative appearance model, muti-peaks target re-detection, scale adaptive, and high-confidence adaptive template updating. The framework of the proposed algorithm is shown in Figure 2. First and foremost, the response fusion of color, HOG, and CN features effectively represent the target appearance. Secondly, a dual-template strategy is presented to promote the discriminant ability of the algorithm. Next, a discriminative appearance model, a multi-peak target re-detection, and a scale adaptive scheme are integrated into the proposed tracker. Finally, the high-confidence adaptive template updating strategy is carried out to enormously decrease the risk of corruption of trackers.

### 4.2. Specific Solution

#### 4.2.1. A Dual-Template Strategy

Due to the limitation of a sole filter template, a dual-template strategy is utilized. According to the kernel form of ridge regression in Equation (11) and the object information given in the first frame, the two filter templates with different sizes *α_b_* and *α_s_* are initialized as:

(15) $${\hat{\alpha }}_{type}=\frac{{\hat{y}}_{type}}{{\hat{k}}_{type}^{xx^{\prime }}+\lambda }\;\;\;type=s\;or\;b,$$

where *α_b_* and *α_s_* represent the big and small templates, respectively. Symbol ^ represents the Fourier transform, *y* is the expected regression label, and *λ* is the regularization parameter. *k^xx^’^^* can be calculated as Equation (12).

In the subsequent frames, assuming that the detection center is the target position of the previous frame and that the size is the small template of the previous frame, the image patch *z* at the same location in the previous frame is intercepted according to the size of the small template *α_s_*. The target region features are extracted and the output response *α_s_* is calculated as Equation (16):

(16) $$f_{type}\left(z\right)=F^{-1}\left({\hat{k}}^{xz}⊙{\hat{\alpha }}_{type}\right)\;\;\;\;\;\;type=s\;or\;b,$$

where the interpretation of *F*^−1^, ⊙ and *k^xz^* is the same as that of Equation (13). *r_s_* and *r_b_* denote the maximum value of *f_s_*(*z*) and *f_b_*(*z*), respectively. The object position of the current frame can be obtained by using the *r_s_* or *r_b_*.

When *r_s_* is bigger than a predefined threshold *Th*, the position corresponding to *r_s_* is taken as the prediction position of the target in the current frame. The small template *α_s_* can accurately predict the target position. When *r_s_* is smaller than a predefined threshold *Th*, we use the big template *α_b_* to calculate response *f_b_*(*z*) according to Equation (16) and can obtain the maximum response value *r_b_*. The final prediction position of the object in the current frame is defined as:

(17) $$p\left(x,y\right)=\{\begin{array}{cc} L\left(f_{s}\left(z\right)\right) & r_{b}\leq r_{s}\\ L\left(f_{b}\left(z\right)\right) & r_{b}>r_{s} \end{array},$$

where *p*(*x*,*y*) is the prediction position of the object in the current frame. The prediction positions *L*(*f_s_*(*z*)) and *L*(*f_b_*(*z*)) are calculated by utilizing the small template and the big template, respectively.

#### 4.2.2. A Discriminative Appearance Model

To effectively distinguish the foreground target from the surrounding background, the proposed tracker uses the Bayesian classifier based on a histogram to establish the color model of the target. For the given foreground target region *O* and its surrounding background region *B*, the probability that the pixel *x* belongs to the region *O* can be computed by:

(18) $$P\left(x\in O|O,B,b_{x}\right)=\frac{P\left(b_{x}|x\in O\right)P\left(x\in O\right)}{\sum_{\Omega \in \left\{O,B\right\}}P\left(b_{x}|x\in \Omega \right)P\left(x\in \Omega \right)},$$

where *b_x_* represents the bin index *b* assigned to the color components of the pixel point *x*. The probabilities $P\left(b_{x}|x\in O\right)$ and $P\left(b_{x}|x\in B\right)$ can be calculated as:

(19) $$\{\begin{array}{c} P\left(b_{x}|x\in O\right)=\frac{H_{O}\left(b_{x}\right)}{\left|O\right|}\\ P\left(b_{x}|x\in B\right)=\frac{H_{B}\left(b_{x}\right)}{\left|B\right|} \end{array},$$

where *H_O_*(*b_x_*) and *H_B_*(*b_x_*) represent the histograms with the range *b* of the regions *O* and *B*, respectively. |O| and |B| represent the areas of foreground target and the background, respectively. Meanwhile, The probabilities P(x∈O) and P(x∈B) can be computed by:

(20) $$\{\begin{array}{c} P\left(x\in O\right)=\frac{\left|O\right|}{\left|O\right|+\left|B\right|}\\ P\left(x\in B\right)=\frac{\left|B\right|}{\left|O\right|+\left|B\right|} \end{array}.$$

Then, Equation (18) can be simplified as:

(21) $$P\left(x\in O|O,B,b_{x}\right)=\frac{H_{O}\left(b_{x}\right)}{H_{O}\left(b_{x}\right)+H_{B}\left(b_{x}\right)}.$$

**Figure 3:**
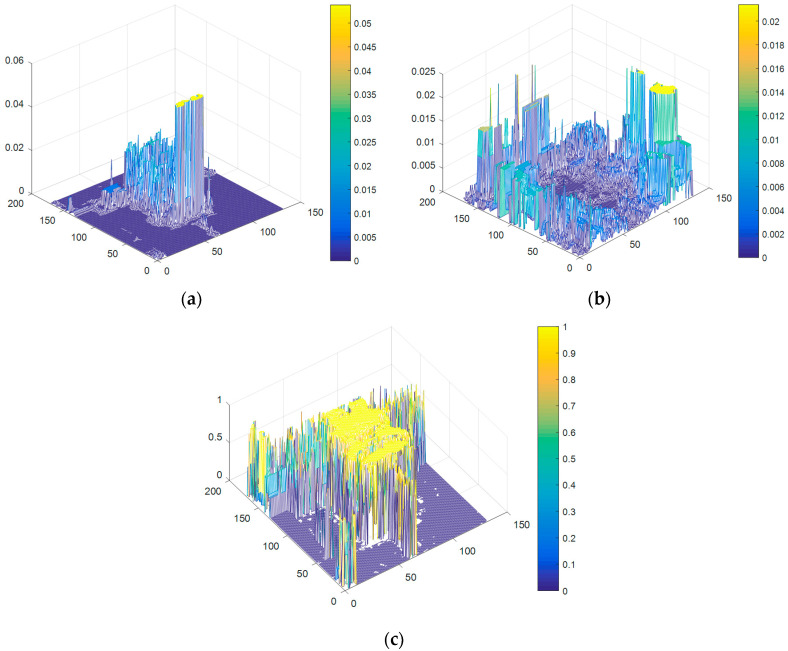
The color histograms of the lemming sequence in frame 138: (**a**) The color histogram of *H_O_*(*b_x_*); (**b**) The color histogram of *H_B_*(*b_x_*); (**c**) The color histogram of $P\left(x\in O|O,B,b_{x}\right)$

For instance, the color histograms of *H_O_*(*b_x_*), *H_B_*(*b_x_*), and $P\left(x\in O|O,B,b_{x}\right)$ of lemming sequence in frame 138 is shown in Figure 3. We can intuitively distinguish the foreground target region *O* and background region *B* by the color histogram of $P\left(x\in O|O,B,b_{x}\right)$.

Subsequently, assuming that the detection center is the predicted target position of the previous frame. According to Equation (21), the probability $P_{t}\left[x\left(i,j\right)\right]$ of the pixel x(i,j) belongs to the target region *O* in frame *t* can be computed. Then, the response of the fore-background color model *f*_color_ can be computed utilizing the average value of an integral image from the $P_{t}\left[x\left(i,j\right)\right]$. The response *f*_color_ can be denoted as:

(22) $$f_{\mathrm{color}}=\frac{1}{\left|M\right|}\sum_{X\left(i,j\right)\in M}P_{t}\left[x\left(i,j\right)\right],$$

where *M* represents the search region centered on the target center position of the previous frame, |•| defines the area of the region.

The tracking performance is sensitive to the changes of scenarios, consequently, we require that the parameters of the tracking model can change adaptively according to the change of the tracking scenarios. To improve the reliability of the tracking model, a feature fusion weight calculation method is utilized by combining peak-to-sidelobe ratio (PSR) and average correlation peak energy (APCE) [45] confidence index to adjust the changes of the challenge scenarios. The PSR is defined as the ratio of the peak intensity of the main lobe to that of the side lobe, which is:

(23) $$PSR=\frac{\max\left(y_{P}\right)-u_{\Phi }\left(y_{P}\right)}{\sigma_{\Phi }\left(y_{P}\right)},$$

where *y_P_* is the confidence map, subscript *Φ* = 0.10 is a constant, *u_Φ_*(·) and *σ_Φ_*(·) are the mean value and standard deviation of the confidence map, respectively.

Next, the APCE is defined as:

(24) $$APCE=\frac{{\left|F_{\max}-F_{\min}\right|}^{2}}{mean\left(\sum_{w,h}{\left(F_{w,h}-F_{\min}\right)}^{2}\right)},$$

where |·| represents the Euclidean distance, *F*_max_, *F*_min_, and *F_w,h_* denote the maximum, minimum, and the *w*-th row *h*-th column elements of the filter response matrix with the size of *W ×* *H*, respectively. The numerator is the square of the difference between the *F*_max_ and the *F*_min_, and the denominator is the square mean of the difference between each element and the minimum value in the filter response matrix. The larger the PSR and APCE, the less occlusion and noise information in the filter template.

In various tracking challenge scenarios, the responses of the two features will change, and the confidence degree will fluctuate to different degrees. In this paper, by combining PSR and APCE, a feature fusion weight strategy is defined as:

(25) $$\{\begin{array}{l} \psi_{i}=\mu_{i}\times PSR_{i}+\upsilon_{i}\times APCE_{i}\\ \zeta_{i}=\frac{\psi_{i}}{\sum_{i}\psi_{i}} \end{array}\;\;\;\;\;i=1,2,3,$$

where *ζ*_1_, *ζ*_2_, and *ζ*_3_ represent the adaptive fusion weight of the color, HOG, and CN features, *ψ*_1_, *ψ*_2_, and *ψ*_3_ represent the confidence factor of the color, HOG and CN features, and *μ*_1_, *μ*_2_, and *μ*_3_ are the PSR control factor of the color, HOG, and CN features, *υ*_1_, *υ*_2_, and *υ*_3_ are the APCE control factor of the color, HOG, and CN features. After calculating the fusion weight of the three features, the final fusion response is obtained by combining the responses of the color, HOG, and CN features. The final fusion response is defined as:

(26) $$f_{\mathrm{fusion}}=\zeta_{1}f_{\mathrm{color}}+\zeta_{2}f_{\mathrm{HOG}}+\zeta_{3}f_{\mathrm{CN}},$$

where *f*_fusion_ represents the final fusion response, *ζ*_1_, *ζ*_2_, and *ζ*_3_ represent the adaptive fusion weight of color, HOG, and CN features. The responses *f*_HOG_ and *f*_CN_ can be calculated by Equation (16), and the response *f*_color_ can be calculated by Equation (22).

**Figure 4:**
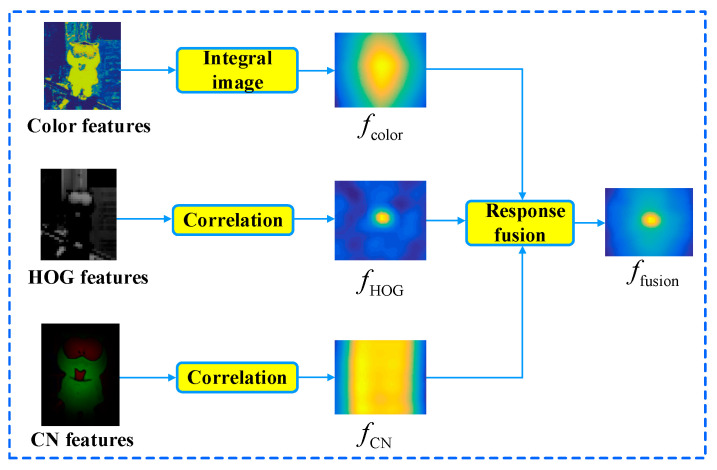
The response fusion of the lemming sequence.

As an example, the response fusion of the lemming sequence is shown in Figure 4, the proposed tracker extracts the color, HOG, and CN features to obtain the response *f*_color_, *f*_HOG_, and *f*_CN_, respectively. The maximum value position of the fusion response *f*_fusion_ is the predicted target center. Therefore, by fusing responses of color, HOG, and CN features, the proposed tracker owns the stronger target representation ability.

#### 4.2.3. A Multi-Peaks Target Re-Detection Technique

In the tracking process, the target may undergo interference of similar appearance, which will lead to inaccurate detection. Meanwhile, partial or full occlusion for a long time may further exacerbate the drift and the corruption of the target appearance model. The disturbance of similar objects and occlusion will fail to track the target successfully. Besides, the multiple peaks perhaps appear in the response map in this interference environment. Therefore, a multi-peaks target re-detection technique is proposed to redetect the tracked target and further improve tracking precision.

First, a spatial weight *w* is presented to computer drastic changes in consecutive frames during the tracking process, and the *w* can be computed by:

(27) $$w^{t}=\exp\left(-\kappa^{t}\left\|C^{t}-C^{t-1}\right\|\right)\;\;,$$

where *C* denotes the predicted target position in the frame *t* and ‖•‖ represents the Euclidean distance, *t* is the frame index. The spatial weight factor κ can be computed as:

(28) $$\kappa =\frac{1}{2*\left({L_{diag}}^{\wedge }2\right)},$$

where *L_diag_* denotes the diagonal length of the target size. The spatial weight *w* is larger, the inter-frame movement is more drastic. In this circumstance, the target may be occluded or interfere with similar objects. When the *w* in frame *t* is less than predefined threshold *Thsw*, the multi-peaks target re-detection technique is activated.

Then, the local maxima locations with the multiple peaks in the fusion response map should be found out. A set of points *M* with these local maxima locations is defined as:

(29) $$M=\left\{\left(i,j\right)|f_{\mathrm{fusion}}\left(i,j\right)>\mu \max\left(f_{\mathrm{fusion}}\right)\right\},$$

where *μ* represents the control parameter.

The responding image patches centered at those local maxima locations are redetected. Subsequently, the maxima response values of the local maxima locations are computed according to Equation (28) and are denoted as a vector score. Besides, the spatial weights of the local maxima locations can be computed by Equation (27) and are denoted as a vector w. The maximum confidence value *multi_peak*_max_ of the local maxima locations can be formulated as:

(30) $$multi\_peak_{\max}=\max(score⊙w),$$

where ⊙ represents the Hadamard product operator. Finally, the final maximum response value *r*_max_ can be obtained by comparing the values of max(*f*_fusion_) and *multi_peak*_max_, which can be written as:

(31) $$r_{\max}=\{\begin{array}{ll} muti\_peak_{\max}, & \max\left(f_{\mathrm{fusion}}\right)<muti\_peak_{\max}\\ \max\left(f_{\mathrm{fusion}}\right), & \max\left(f_{\mathrm{fusion}}\right)\geq muti\_peak_{\max} \end{array}.$$

The optimal target position can be estimated by the corresponding location of *r*_max_.

#### 4.2.4. A Scale Adaptive Scheme

Owing to the drawbacks of the fixed search range of the object, we employ a scale adaptive scheme. To appraise the scale size of the object, the one-dimensional scale filter described in [46] is utilized to solve the scale problem. Firstly, the scale filter *F_d_* is defined as:

(32) $$\begin{array}{ll} F_{scale}^{d} & =\frac{Y_{scale}⊙{\left(X_{scale}^{d}\right)}^{*}}{\sum_{d=1}^{D}X_{scale}^{d}⊙{\left(X_{scale}^{d}\right)}^{*}+\lambda^{\prime }}\\ & =\frac{A_{scale}^{d}}{B_{scale}+\lambda^{\prime }}, \end{array}$$

where *X_scale_* and *Y_scale_* are the Fourier transform of the training sample *x_scale_* and the expected regression label *y_scale_*, respectively. *D* is the channel number of feature maps *x_scale_*, and *λ’* is the regularization parameter. Then, after estimating the object position, we extract feature maps *z_scale_* from the new predicted position of the object. The correlation response *f_scale_*(*z*) is computed by:

(33) $$f_{scale}\left(z\right)=F^{-1}\left\{\frac{\sum_{d=1}^{D}{\left(A_{scale}^{d}\right)}^{*}⊙Z_{scale}^{d}}{B_{scale}+\lambda^{’}}\right\},$$

where *Z_scale_* is the Fourier transform of the test sample *z_scale_*. The maximum value of *f_scale_*(*z*) is the object scale. Then, to robustly learn the scale filter, the filter model is updated as:

(34) $$\{\begin{array}{l} A_{scale,t}^{d}=\left(1-\eta_{scale}\right)A_{scale,t-1}^{d}+\eta_{scale}Y_{scale}⊙{\left(X_{scale,t}^{d}\right)}^{*}\\ B_{scale,t}=\left(1-\eta_{scale}\right)B_{scale,t-1}+\eta_{scale}\sum_{d=1}^{D}X_{scale,t}^{d}⊙{\left(X_{scale,t}^{d}\right)}^{*} \end{array},$$

where *η_scale_* is a learning rate parameter, and the numerator $A_{scale,t}^{d}$ and $A_{scale,t-1}^{d}$ are the *d*-th channel filter template in the frames *t* and *t*-1, respectively. Besides, the denominators *B_scale,t_* and *B_scale,t-1_* are the templates in the frames *t* and *t*-1, respectively. The scale adaptive scheme further enhances the robustness of the tracker.

#### 4.2.5. A High-Confidence Template Updating Technique

To further improve the reliability of the tracking model, APCE is taken as a judgment basis of whether the filter model needs to be updated. Because APCE measures the fluctuation degree of the response map, when the target is appearing in the search region, APCE will become larger, indicating that the tracking model almost has no disturbance. Otherwise, if the target is encountering challenges (e.g., occlusion or the interference of similar objects), APCE will become smaller and the fluctuation degree of the response map is fierce. In the situation of occlusion or the interference of similar objects, the filter model should not be updated.

**Figure 5:**
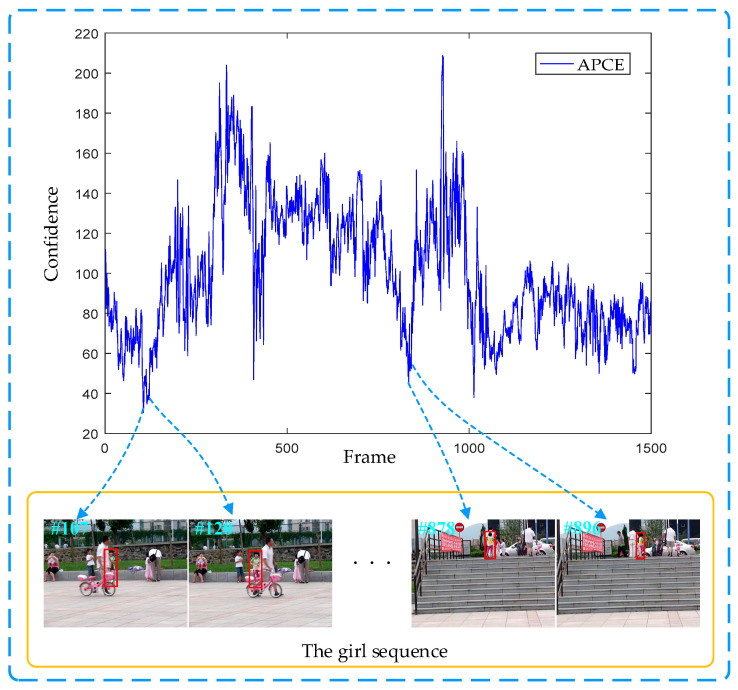
An example of the average correlation peak energy (APCE) being sensitive to the visual tracking environment on the girl sequence in the VOT2016 dataset.

An example of the APCE being sensitive to the visual tracking environment on the girl sequence is shown in Figure 5. For instance, we notice that APCE drops rapidly when occlusion occurs at frame 107. When the occlusion disappears at frame 120, APCE begins to increase. Subsequently, APCE drops rapidly when the target is disturbed by a similar object of the surrounding background in frame 878. Then, APCE begins to increase when the distractor disappears at frame 896 in the video sequence. According to the criteria in [41], the template will be not updated until the occlusion or similar object disappears. The evaluation criterion of the high-confidence template updating is given as:

(35) $$\Omega =\{\begin{array}{cc} 1, & if\;APCE_{t}>\frac{\sum_{i=t-m}^{t-1}APCE_{i}}{m}\times \xi \\ 0, & else \end{array},$$

where *APCE_t_* is the APCE of the frame *t*, proportion factor *ξ* is a constant, *Ω* means the control coefficient of model updating. Specifically, if *Ω* = 0, it illustrates that the confidence degree of the model is low and the object is occluded or missing, the model will not be updated. If *Ω* = 1, it illustrates that the confidence degree of the model is high, and the updating of small and big templates can be mathematically expressed as:

(36) $${\hat{\alpha }}_{type}^{t}=\left(1-\Omega \cdot \tau \varepsilon_{t}\right){\hat{\alpha }}_{type}^{t-1}+\Omega \cdot \tau \varepsilon_{t}{\hat{\alpha }}_{type}^{new}\;\;\;\;\;type=s\;or\;b,$$

where *ε_t_* = *APCE_t_* is the learning rate parameter in frame *t*, and *τ* is the control factor.

## 5. Experimental Results and Analysis

In this section, a comprehensive experimental evaluation of various trackers is carried out. The specific process description is provided below.

### 5.1. Experiment Setup

The native MATLAB R2016b without optimization was utilized to implement the proposed tracking algorithm. The experiments were performed on an Intel (R) Core (TM) i7-9750H CPU (2.60 GHz) laptop with 16 GB main memory. The initial position and size of the target based on a bounding box centered on the target in the first frame were given in advance. The HOG and CN features were employed in the proposed tracking algorithm. In this paper, the tracking benchmark databases OTB2013, OTB2015, VOT2016, and LaSOT [47] were utilized for simulation experiments. In the proposed algorithm, in the fore-background color model, the color features were RGB and the number of histogram bins was 32. The cell size of the HOG features was 4 × 4 and the channel number of HOG features was 31. Then, the channel number of CN was 11. The spatial regularization parameter *λ* was set to 0.01. The small and large templates adopted 1.2- and 2.5-times the object size, respectively. The dual-template threshold was set to 0.65. In the scale filter, the scale level *S* was set to 33, the scale factor *a* was set to 1.02, and the feature of each scale level used the feature vector, which was obtained by 31-*D* HOG features, the learning rate of the scale filter was *η_scale_* = 0.025. The proportion factor *ξ* was set to 0.707, and the control factor *τ* was set to 0.0003. Furthermore, the same parameter values and initialization were utilized for all the video sequences.

### 5.2. Compared Trackers

In order to comprehensively evaluate the proposed tracking algorithm, 27 state-of-the-art trackers were introduced for comparison purposes. These trackers can be divided into two categories: (a) 16 state-of-the-art handcrafted features-based trackers, including AO-CF, MMWCF, spatial-temporal regularized correlation filters (STRCF) [48], parallel tracking and verifying (PTAV) [49], channel and spatial reliability correlation filter trackers (CSRDCF) [50], fDSST, spatially regularized correlation filters based on adaptive decontamination (SRDCFdecon) [51], sum of template and pixel-wise learners (Staple) [52], EdgeBox tracker (EBT), LCT, SRDCF, KCF, scale adaptive with multiple features tracker (SAMF) [53], DSST, CN, and CSK, (b) 11 deep features-based trackers including SPLT, siamese instance search tracker (SINT) [54], efficient convolution operators (ECO) [55], continuous convolution operator tracker (CCOT) [56], MDNET, SiamFC, structured siamese network (StructSiam) [57], dynamic siamese network (DSiam) [58], tracker based on context-aware deep feature compression with multiple auto-encoders (TRACA) [59], end-to-end representation learning for correlation filter (CFNet) [60], and HCF.

### 5.3. Experimental Results on the OTB2013 and OTB2015 Benchmark Databases

To gain more insights into the effectiveness of the proposed tracker, we evaluated and analyzed the overall performance, attribute-based evaluation, and qualitative comparison of ours and the other 12 trackers on the OTB2013 and OTB2015 benchmark databases. In the experiment, we compared our tracker against nine state-of-the-art HFTs: CSK, CN, DSST, SAMF, KCF, Staple, SRDCFdecon, fDSST, and AO-CF. Then, three DFTs, ECO, CCOT, and HCF, were also compared with our tracker. 

#### 5.3.1. The OTB2013 and OTB2015 Benchmark Databases

The OTB2013 database has 51 video sequences. Subsequently, the OTB2015 tracking benchmark database was expanded to 100 video sequences, even with some long sequences.

**Figure 6:**
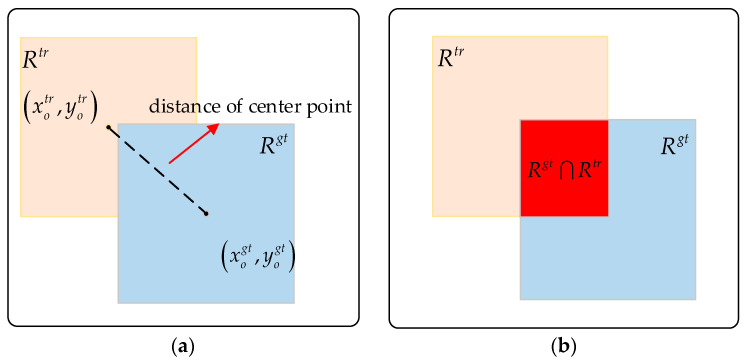
Evaluation criterion. (**a**) Distance of the center point. (**b**) Intersection over the union.

The OTB2015 dataset evaluates tracking algorithms in two aspects: precision plot and success plot. As shown in Figure 6, the precision plot represents different distance precisions (DP) under different thresholds for the target center location error (CLE), where CLE is the average Euclidean distance between the target prediction center location and the ground truth. DP is computed as the percentage of frames in the videos where the CLE is smaller than 20 pixels. The success plot represents different average overlap precisions (OP) under different thresholds for the overlap success score *S*, where *S* = |*R^tr^*∩*R^gt^*|/|*R^tr^*∪*R^gt^*|, *R^tr^* denotes the prediction box, *R^gt^* denotes the ground truth, ∩ and ∪ denote the intersection and union of two areas, and |·| denotes the number of pixels in the area. OP is the percentage of frames where the overlap between the prediction box and the ground truth exceeds 0.5. The area under the curve (AUC) was computed to evaluate the proposed tracker’s performance.

#### 5.3.2. Overall Performance Evaluation

**Figure 7:**
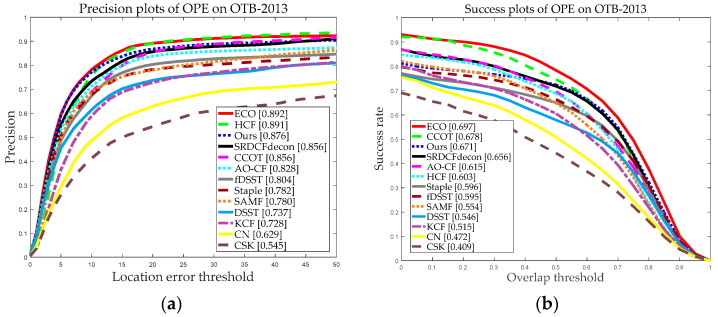
Precision and success plots on the dataset OTB-2013 using one-pass evaluation (OPE). (**a**) Precision plots of OPE on OTB-2013; (**b**) Success plots of OPE on OTB-2013. In the legends, the average distance precisions (DP) rates at a threshold of 20 pix and area under the curve (AUC) scores at a threshold of 0.5 are reported for the precision plots and success plots, respectively.

**Figure 8:**
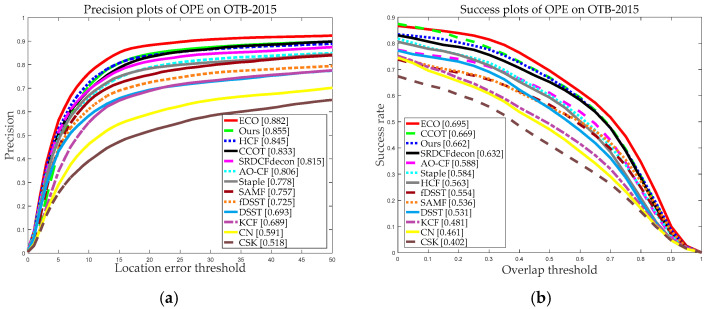
Precision and success plots on the dataset OTB-2015 using OPE. (**a**) Precision plots of OPE on OTB-2015; (**b**) Success plots of OPE on OTB-2015. In the legends, the average DP rates at a threshold of 20 pix and AUC scores at a threshold of 0.5 are reported for the precision plots and success plots, respectively.

Figure 7 and Figure 8 demonstrate the graphical performance results of our and the other 12 trackers using the two datasets. As we can see from Figure 7 and Figure 8, our tracker achieved precision scores of 0.876 and 0.855 and success scores of 0.671 and 0.662 for the OTB-2013 and OTB-2015 datasets, and the precision scores and success scores of our tracker ranked third of all 13 trackers. Compared with the classical KCF, our tracker made about 21.77% and 38.67% improvement in terms of precision scores and success scores on the OTB-2015 dataset. Besides, from Figure 7 and Figure 8, we can see that our tracker performed favorably against nine state-of-the-art trackers with handcrafted features, compared with the ECO, CCOT, and HCF based on deep features, our tracker achieved comparable results. The running speed is also an important evaluation index for measuring the performance of the trackers. Table 1 and Table 2 demonstrate the average tracking speed (FPS) comparison on OTB2013 and OTB2015. Particularly, due to the large computation overhead and complexity, the average FPS of ECO, CCOT, and HCF was approximately 1 FPS. Overall, the ECO, CCOT, and HCF performed well in terms of precision scores and success scores but at a lower tracking speed. Our tracker achieved an average tracking speed of 21.536 and 20.103 FPS on the OTB2013 and OTB2015, respectively, which is significantly faster than DFTs. Meanwhile, our tracker can meet real-time requirements.

#### 5.3.3. Attribute-Based Evaluation

**Figure 9:**
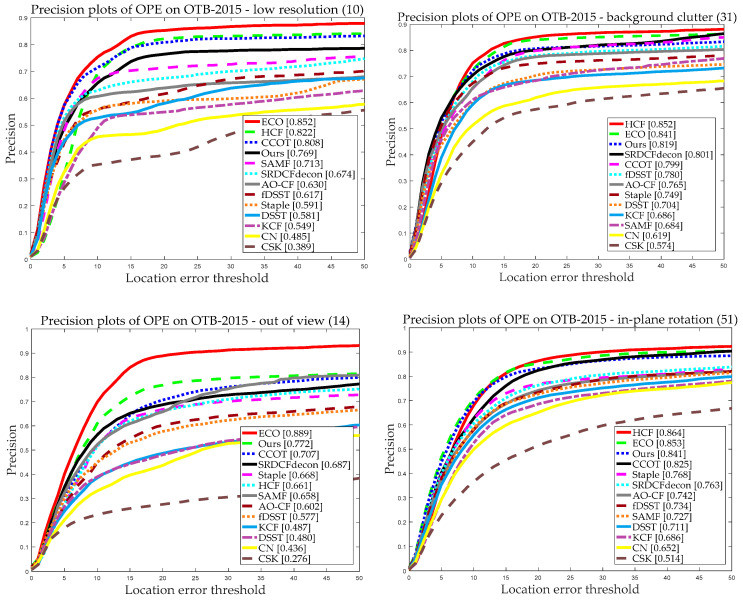

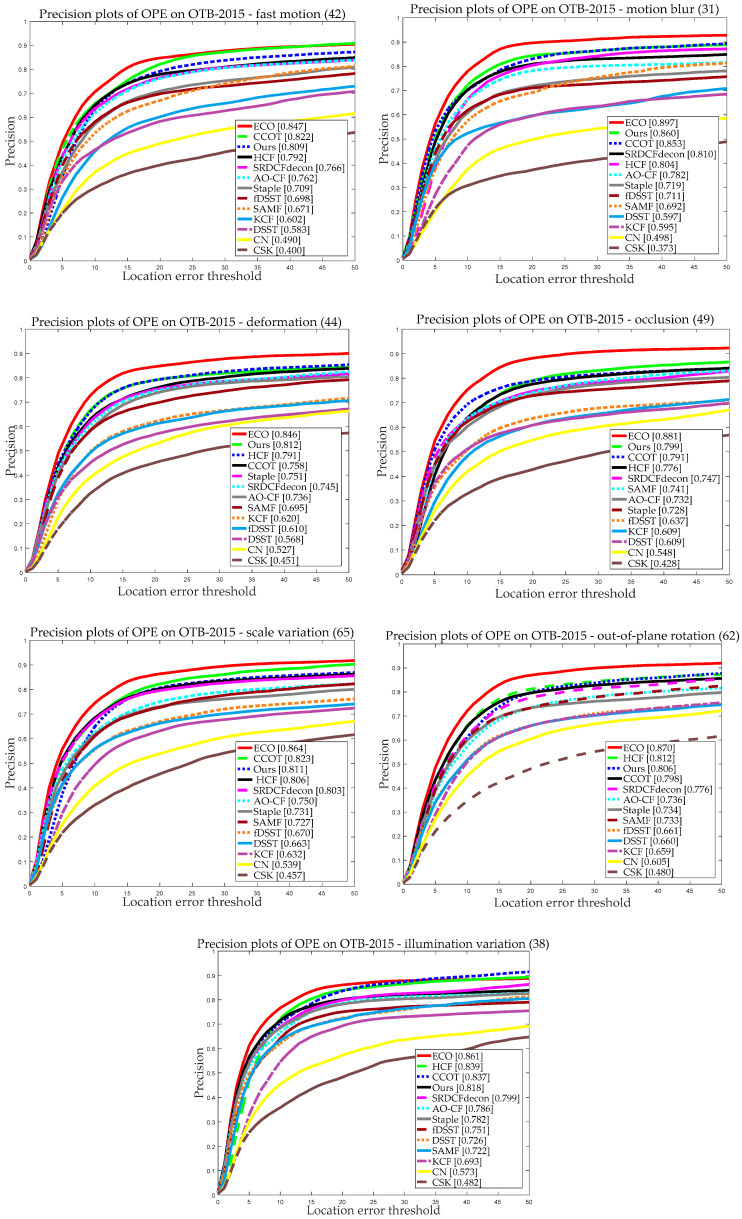
Precision plots for the compared trackers on OTB-2015 with 11 attributes using OPE.

In the two datasets, the 11 diverse challenging scenarios (as shown in Figure 1a) significantly affected the performance of the trackers. As these challenging scenarios come up from time to time, it may lead to the corruption of the filter model and eventually to track failure. Figure 9 demonstrates the attribute-based precision evaluation of our and the other 12 trackers using the OTB2015 dataset. As we can see from Figure 9, in terms of precision plots, our tracker ranked second for 4 out of the 11 challenging scenarios and ranked third for 5 out of the 11. Therefore, among all 11 challenging scenarios, our tracker achieved a comparative tracking performance. Compared with three DFTs, the performance of our tracker was comparable in most cases.

#### 5.3.4. Qualitative Comparison

**Figure 10:**
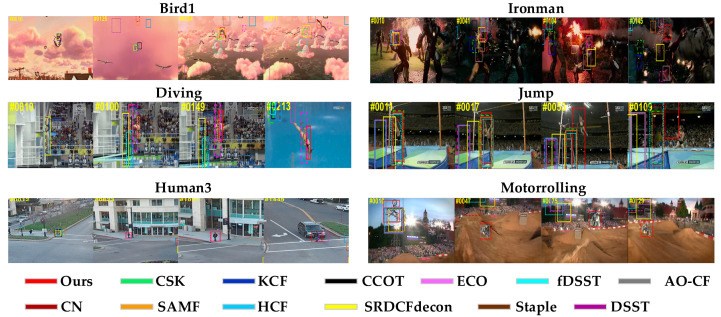
Qualitative evaluation of six video sequences (i.e., Bird1, Ironman, Diving, Jump, Human3, and Motorrolling). We show the results of ours and the other 12 trackers, including nine HFTs and three DFTs with different colors (our results are in red).

In a comparative experiment, to better show the tracking performance, Figure 10 demonstrates the qualitative evaluation results of our, the other nine HFTs, and three DFTs. The frames from six representative video sequences with Bird1, Ironman, Diving, Jump, Human3, and Motorrolling were illustrated. As shown in Figure 10, at the beginning of video sequence frames, most tracking algorithms could keep up with the target. The targets of the Bird1 and Ironman video sequences mainly undergo the challenges of fast motion and motion blur, some trackers may lose target or pull-in unnecessary background information due to the fixed detection range, our approach can track the target successfully by utilizing the dual-template method. The main challenge of the Human3 and Diving video sequences is scale change; some trackers without scale estimation cannot deal with this scenario well, and the proposed method can overcome the limitation of large scale variations by using the scale adaptive scheme. Jump and Motorrolling video sequences are accompanied by occlusion and deformation, which may cause some trackers to drift; our algorithm can deal with occlusion better owing to the adaptive template updating. In general, the proposed approach can keep track of the target in the whole video sequence.

### 5.4. Experimental Results on the VOT2016 Benchmark Dataset

To gain more insights into the effectiveness of the proposed algorithm, we further exhibited the quantitative and qualitative comparison on the VOT2016 database. In the experiment, we compared our tracker against nine state-of-the-art HFTs (CN, DSST, SAMF, SRDCF, KCF, Staple, EBT, SRDCFdecon, and MMWCF) and three DFTs (SiamFC, MDNET, and HCF).

#### 5.4.1. The VOT2016 Benchmark Database

The VOT2016 database consists of 60 challenging sequences and is relatively a difficult sequence set in contrast to OTB2015. The VOT2016 dataset has five common video challenge scenarios: camera motion, illumination variation, motion change, occlusion, and size change. Two basic tracking evaluation indexes (i.e., accuracy and robustness) were utilized in this work. The accuracy denotes the average overlap rate of the tracking success state, while the robustness was measured by the total number of tracking failures. Additionally, the values of per-frame accuracy and robustness were combined as the expected average overlap (EAO) to measure the overall performance.

#### 5.4.2. Quantitative and Qualitative Comparison

**Figure 11:**
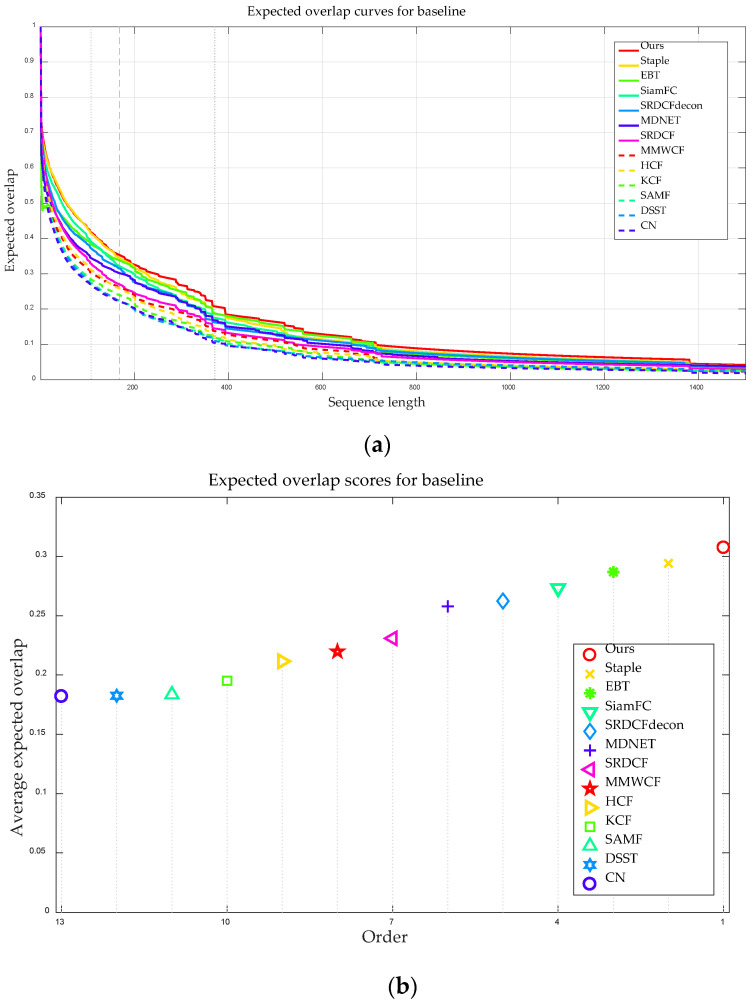
Comparison of the expected average overlap (EAO) for different trackers. (**a**) Ranking results of EAO, where the better trackers are located at the top and right. (**b**) EAO curves of trackers over different sequences lengths.

To further assess the effectiveness and accuracy of our tracker, Figure 11 demonstrates the comparison result of EAO under the different trackers. Figure 11a shows the ranking results of EAO and Figure 11b exhibits the EAO curves of 13 trackers over different sequences lengths. From Figure 11, we can see that the proposed tracker obtained an EAO score of 0.308 and ranked first among all the trackers. Table 3 presents the EAO score of each approach and the best three trackers are shown in red, blue, and green bold fonts. Particularly, compared with the Staple and EBT approach, our tracker made about 4.55% and 5.84% improvement, respectively. Table 4 demonstrates the accuracy evaluation of ours and other 12 state-of-the-art trackers on different challenging attributes. As shown in Table 4, our tracker obtained a significant gain in accuracy and achieved the top performer except for the camera motion and motion change. Overall, our approach performed better than the nine HFTs and three DFTs.

**Figure 12:**
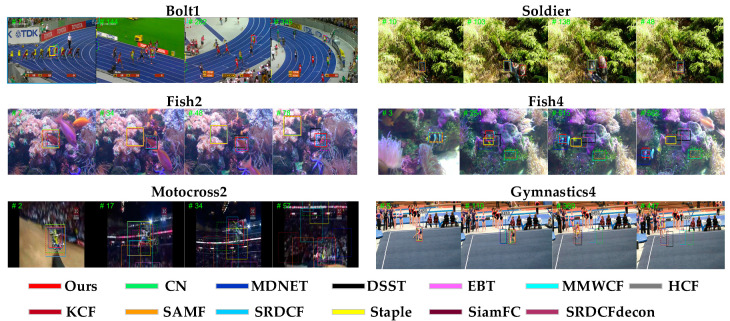
Qualitative evaluation on six video sequences (i.e., Bolt1, Soldier, Fish2, Fish4, Motocross2, and Gymnastics4) in the unsupervised experiment. We show the results of ours and the other 12 trackers, including 9 handcrafted features-based trackers and 3 deep features-based trackers with different colors (our results are in red).

Figure 12 shows qualitative evaluation results of our and the other 12 trackers on six video sequences, i.e., Bolt1, Soldier, Fish2, Fish4, Motocross2, and Gymnastics4. For instance, the Bolt1 and Soldier video sequences experience size change from large to small. When the object becomes smaller and the search range is the same, it will bring in considerably background noises and disturbance and make the drift; when the object becomes larger, the search range can only contain the local information of the object. Therefore, the fixed search range of object pollutes the object template and reduces the robustness of the tracker. The proposed approach can deal with size change well by the dual-template method and scale adaptive scheme. The Fish2 and Fish4 video sequences have the scenarios of moving swiftly or large movement range; due to the small detection range, most trackers will not be able to catch up with the target or even lose the target. Our tracker can track the object successfully due to the dual-template method. The Motocross2 and Gymnastics4 video sequences are accompanied by motion change and occlusion. The parameters of the tracking model are generally set as a fixed value in many trackers, which may cause the models to drift or even fail. Nevertheless, our algorithm can deal with the challenges due to the high-confidence adaptive template updating. As shown in Figure 12, the experimental results demonstrate that the proposed algorithm had the best tracking performance for the six video sequences.

### 5.5. Experimental Results on the LaSOT Benchmark Dataset

The LaSOT dataset is a high-quality long-term tracking dataset, which is much closer to realistic applications. The LaSOT dataset contains 1400 video sequences with more than 3.5M frames in all, and the average video length of the dataset is more than 2500 frames. Additionally, the number of object categories in LaSOT dataset is 70, which is two times more than the existing dense benchmark. The LaSOT dataset has 14 video challenges (i.e., aspect ratio change, deformation, fast motion, full occlusion, viewpoint change, and out-of-view). Besides, the most challenges of LaSOT dataset derive from the wild. During the tracking process, the trackers will encounter many complicated challenge scenarios. Similar to the OTB2013 and OTB2015 datasets, the LaSOT dataset evaluates tracking algorithms with precision and success plots.

**Figure 13:**
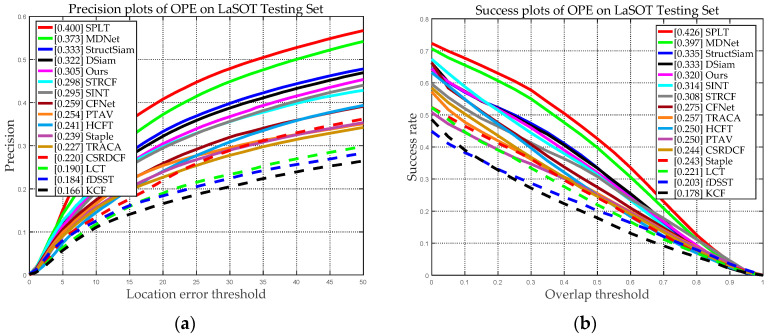
Precision and success plots on LaSOT using OPE. (**a**) Precision plots of OPE on LaSOT; (**b**) Success plots of OPE on LaSOT.

In the experiment, we compared our tracker with the seven recent HFTs (LCT, KCF, STRCF, PTAV, Staple, fDSST, and CSRDCF) and eight DFTs (SPLT, MDNet, SINT, StructSiam, DSiam, TRACA, CFNet, and HCFT). Figure 13 shows the overall performance results of our and the other 15 state-of-the-arts tracking approaches using LaSOT dataset. As we can see from Figure 13, our approach obtained a precision score of 0.305 and a success score of 0.320, and the precision and success scores of the proposed tracker ranked fifth in total. Compared with the top four tracking algorithms (SPLT, MDNet, StructSiam, and DSiam), our tracking approach still has an accuracy gap. Compared with the top four deep learning-based tracking algorithms (SPLT, MDNet, StructSiam, and DSiam), our tracking approach still has an accuracy gap. However, with the increase in the network layers number of the deep learning-based trackers, the computational complexity and parameter storage space are increased exponentially. Usually, trackers are used in monitoring or removable embedded devices, which have limited computing power and storage and cannot meet the requirements of deep learning-based trackers. On the contrary, the tracking accuracy of our tracker can meet the needs of many applications and can be realized on embedded devices. Meanwhile, compared with the seven classical handcrafted features-based trackers, our tracker obtained a significant performance improvement. For instance, compared with LCT, which is a traditional long-term tracker, our approach made about 37.70% and 30.94% improvement in terms of precision and success scores.

## 6. Conclusions and Discussion

Object tracking has been a hot research topic in various fields. However, in the face of complicated challenges, the performance of the trackers still needs to be improved. In this paper, a novel dual-template CFT for various typical challenge scenarios was investigated. Different from the traditional tracking approach, the proposed tracker employs the strategies of dual-template strategy, discriminative appearance model, multi-peak target re-detection, scale adaptive scheme, and adaptive filter template updating, which can promote the effectiveness and robustness of the tracking approach. Meanwhile, 27 existing competitors, including 16 HFTs and 11 DFTs, were introduced for the comprehensive contrastive analysis on the OTB2013, OTB2015, VOT2016, and LaSOT benchmark databases. The experimental results show that the proposed tracker performs favorably against state-of-the-art HFTs and is comparable with the DFTs. In the future, we are considering the combination of a deep learning framework to further improve the accuracy and robustness of the proposed algorithm. To effectively track the target on a large-scale and long-term video dataset, we will further mine and study the re-detection mechanism and search region strategy.

## Figures and Tables

**Table 1:** The speeds in comparison of different trackers on the OTB-2013 dataset.

	Ours	ECO	CCOT	HCF	fDSST	SRDCFdecon	Staple	AO-CF	SAMF
Avg.FPS	21.536	1.271	0.524	0.740	71.079	2.203	83.484	52.107	18.469

**Table 2:** The speeds in comparison of different trackers on the OTB-2015 dataset.

	Ours	ECO	CCOT	HCF	fDSST	SRDCFdecon	Staple	AO-CF	SAMF
Avg.FPS	20.103	1.245	0.407	0.720	66.353	2.195	78.356	48.936	16.120

**Table 3:** The EAO performance comparison of ours and seven other state-of-the-art trackers on the VOT2016 dataset. The best two results are marked in red and blue bold fonts.

	Ours	SiamFC	EBT	MDNET	HCF	SRDCFdecon	Staple	SRDCF
EAO	**0.308**	0.277	0.290	0.258	0.220	0.262	**0.294**	0.231

**Table 4:** Accuracy evaluation of ours and seven other state-of-the-art trackers on different challenging attributes of the VOT 2016 benchmark database. The best two results are marked in red and blue bold fonts, respectively.

	Ours	SiamFC	EBT	MDNET	HCF	SRDCFdecon	Staple	SRDCF
Camera motion	**0.560**	**0.563**	0.491	0.547	0.438	0.530	0.551	0.551
Illumination change	**0.718**	0.672	0.407	0.639	0.462	**0.714**	0.709	0.680
Motion change	**0.528**	**0.530**	0.439	0.508	0.423	0.466	0.507	0.486
Occlusion	**0.499**	0.448	0.375	**0.491**	0.433	0.434	0.433	0.408
Size change	**0.528**	**0.514**	0.356	0.511	0.354	0.490	0.511	0.478
Empty	**0.597**	**0.586**	0.518	0.563	0.502	0.524	0.584	0.580
Mean accuracy	**0.572**	**0.552**	0.431	0.543	0.435	0.526	0.549	0.530
Weighted mean accuracy	**0.563**	**0.549**	0.453	0.537	0.437	0.509	0.540	0.523
